# The Cyclopedia of Practical Quotations

**Published:** 1884-11

**Authors:** 


THE CYCLOPEDIA OF PRACTICAL QUOTATIONS, English and Latin, with an Appendix, containing Proverbs from Latin and Modern Foreign Languages; Law and Ecclesiastical Terms and Significations; Names, Dates and Nationality of Noted Authors, etc., with Copious Indices. By J. K. Hoyt and Anna L. Ward. Sixth Edition. Funk & Wagnalls, Publishers, 10 and 12 Dey street, New York. 1884.

The authors and publishers of this work are to be congratulated upon the completion of a book that must have cost almost a lifetime of labor. We have carefully examined and tested it, and find it indeed a valuable reference book. It is indispensable to every one who may have frequent occasions to look up quotations.

